# Establishment of a novel anti-TROP2 monoclonal antibody TrMab-29 for immunohistochemical analysis

**DOI:** 10.1016/j.bbrep.2020.100902

**Published:** 2021-01-12

**Authors:** Yusuke Sayama, Mika K. Kaneko, Junko Takei, Hideki Hosono, Masato Sano, Teizo Asano, Yukinari Kato

**Affiliations:** a Department of Antibody Drug Development, Tohoku University Graduate School of Medicine, 2-1 Seiryo-machi, Aoba-ku, Sendai, Miyagi, 980-8575, Japan; b New Industry Creation Hatchery Center, Tohoku University, 2-1 Seiryo-machi, Aoba-ku, Sendai, Miyagi, 980-8575, Japan

**Keywords:** TROP2, CBIS method, Monoclonal antibody, Breast cancer, ADC, antibody-drug conjugates, ADCC, antibody-dependent cellular cytotoxicity, BSA, bovine serum albumin, CAR-T, chimeric antigen receptor T-cell, CBIS, Cell-Based Immunization and Screening, CDC, complement-dependent cytotoxicity, CHO, Chinese hamster ovary, DAB, 3,3′-diaminobenzidine tetrahydrochloride, mAb, monoclonal antibody, P3U1, P3X63Ag8U.1, PBS, phosphate-buffered saline, PIT, photoimmunotherapy, RIT, radioimmunotherapy, TROP2, trophoblast cell-surface antigen, PVDF, polyvinylidene difluoride

## Abstract

TROP2 is a type I transmembrane glycoprotein originally identified in human trophoblast cells that is overexpressed in several types of cancer. To better understand the role of TROP2 in cancer, we herein aimed to develop a sensitive and specific anti-TROP2 monoclonal antibody (mAb) for use in flow cytometry, Western blot, and immunohistochemistry using a Cell-Based Immunization and Screening (CBIS) method. Two mice were immunized with N-terminal PA-tagged and C-terminal RAP/MAP-tagged TROP2-overexpressed Chinese hamster ovary (CHO)–K1 cells (CHO/PA-TROP2-RAP-MAP), and hybridomas showing strong signals from PA-tagged TROP2-overexpressed CHO–K1 cells (CHO/TROP2-PA) and weak-to-no signals from CHO–K1 cells were selected using flow cytometry. We demonstrated using flow cytometry that the established anti-TROP2 mAb, TrMab-29 (mouse IgG_1_ kappa), detected TROP2 in MCF7 breast cancer cell line as well as CHO/TROP2-PA cells. Western blot analysis showed a 40 kDa band in lysates prepared from both CHO/TROP2-PA and MCF7 cells. Furthermore, TROP2 was strongly detected by immunohistochemical analysis using TrMab-29, indicating that TrMab-29 may be a valuable tool for the detection of TROP2 in cancer.

## Highlights

• TROP2 is overexpressed in several types of cancers.
• We established a novel anti-TROP2 mAb (TrMab-29) using CBIS method.
• TrMab-29 is useful for flow cytometry, western blot, and immunohistochemical analyses.
• TrMab-29 may be a valuable tool for the detection of TROP2 in cancer.

## 1 Introduction

The trophoblast cell-surface antigen (TROP2) is a type I transmembrane glycoprotein that was originally identified in human trophoblast cells [[1], [2], [3]]. TROP2 is highly expressed in many cancers and may play a critical role in tumor progression [4,5]. Because increased TROP2 expression has been reported in more than 85% of all solid cancers, TROP2 may be a useful marker for cancer diagnosis and immunotherapy [[6], [7], [8]]. It has also been identified in stem cells of various tissues, including basal cells, all of which are capable of self-renewal, regeneration, and differentiation [6,9,10]. Further, several monoclonal antibodies (mAbs) against TROP2 are currently evaluated in clinical cancer trials, including DS-1062a [4,11], PF-06664178 [4,12], and IMMU-132 [4,13,14].

We previously established a Cell-Based Immunization and Screening (CBIS) method, in which cell lines are used exclusively for both immunization and screening [15]. CBIS has been employed to develop sensitive and specific mAbs against many transmembrane proteins, including CD19 [16], CD20 [17], CD44 [18], CD133 [15], PD-L1 [19], and podoplanin [[20], [21], [22], [23], [24], [25], [26]]. Importantly, those mAbs have proven to be useful in flow cytometry, Western blot, and immunohistochemical analyses. In this study, we developed a novel anti-TROP2 mAb (TrMab-29) and evaluated its usefulness in flow cytometry, Western blot, and immunohistochemical analyses.

## 2 Materials and methods

### 2.1 Plasmid preparation

Human TROP2 DNA was synthesized commercially by Thermo Fisher Scientific, Inc. (Waltham, MA, USA). TROP2 DNA with an N-terminal PA tag [27] and a C-terminal RAP tag [28]/MAP tag [29] (PA-TROP2-RAP-MAP) was subcloned into a pCAG-Ble expression vector (FUJIFILM Wako Pure Chemical Corporation, Osaka, Japan) using an In-Fusion HD Cloning Kit (Takara Bio, Inc., Shiga, Japan) according to the manufacturer's instructions; the recombinant expression vector was named pCAG/PA-TROP2-RAP-MAP. TROP2 DNA with a C-terminal PA tag was also subcloned into a pCAG-Ble vector using an In-Fusion HD Cloning Kit (Takara Bio, Inc.); this expression vector was named pCAG/TROP2-PA. The amino acid sequences of each tag are as follows: PA tag, consisting of 12 amino acids (GVAMPGAEDDVV); RAP tag, 12 amino acids (DMVNPGLEDRIE); and MAP tag, 12 amino acids (GDGMVPPGIEDK).

### 2.2 Cell lines

MCF7 was obtained from the Cell Resource Center for Biomedical Research, Institute of Development, Aging and Cancer, Tohoku University (Miyagi, Japan). Chinese hamster ovary (CHO)–K1, P3X63Ag8U.1 (P3U1), Lec1, Lec2, and Lec8 cell lines were obtained from the American Type Culture Collection (Manassas, VA, USA).

CHO–K1 cells overexpressing TROP2-PA (CHO/TROP2-PA) and PA-TROP2-RAP-MAP (CHO/PA-TROP2-RAP-MAP) were established by transfecting pCAG/TROP2-PA and pCAG/PA-TROP2-RAP-MAP in CHO–K1 cells, respectively, using Lipofectamine LTX Reagent (Thermo Fisher Scientific, Inc.) according to the manufacturer's protocol. The transfected cells were selected by limiting dilution in the medium containing 0.5 mg/mL of zeocin (InvivoGen, San Diego, CA, USA). The transfected cells were confirmed as TROP2-positive by flow cytometry (EC800, Sony Corp., Tokyo, Japan) using an anti-TROP2 antibody (Cat#LS-C489657, LS Bio, Seattle, WA, USA).

Lec1/TROP2, Lec2/TROP2, and Lec8/TROP2 were established by transfecting pCAG/TROP2-PA to Lec1, Lec2, and Lec8 cells, respectively, using the Neon Transfection System (Thermo Fisher Scientific, Inc.). Stable transfectants were established by cell sorting using SH800 (Sony Corp.), and were cultivated in the medium containing 0.5 mg/mL of zeocin (InvivoGen).

The TROP2-knockout cell line, MCF7/TROP2-KO (BINDS-29), was established by transfecting CRISPR/Cas9 plasmids for TROP2 into MCF7 cells using the Neon Transfection System (Thermo Fisher Scientific, Inc.). Stable transfectants were established by cell sorting using SH800 (Sony Corp.).

CHO–K1, CHO/TROP2-PA, CHO/PA-TROP2-RAP-MAP, P3U1, MCF7, Lec1/TROP2, Lec2/TROP2, Lec8/TROP2, and BINDS-29 cells were cultured in Roswell Park Memorial Institute (RPMI) 1640 medium (Nacalai Tesque, Inc., Kyoto, Japan) supplemented with 10% heat-inactivated fetal bovine serum (Thermo Fisher Scientific, Inc.), 100 U/mL penicillin, 100 μg/mL streptomycin, and 0.25 μg/mL amphotericin B (Nacalai Tesque, Inc.). Cells were grown in a humidified incubator at 37 °C with 5% CO_2_.

### 2.3 Hybridoma production

Female BALB/c mice (6 weeks old) were purchased from CLEA Japan (Tokyo, Japan) and kept under specific pathogen-free conditions. All animal experiments were conducted in accordance with relevant guidelines and regulations to minimize animal suffering and distress in the laboratory. The Animal Care and Use Committee of Tohoku University approved all animal experiments performed in this study (Permit number: 2019NiA-001). Mice were euthanized by cervical dislocation under anesthesia, and death was verified by respiratory and cardiac arrest. We employed CBIS to develop new mAbs against TROP2. Two mice were immunized with CHO/PA-TROP2-RAP-MAP cells (1 × 10^8^ cells/mouse) via intraperitoneal (i.p.) injection with Imject Alum (Thermo Fisher Scientific, Inc.). After several additional immunizations, a booster immunization was administered by i. p. injection 2 days before spleen cells were collected. The collected spleen cells were fused with P3U1 cells using polyethylene glycol 1500 (Roche Diagnostics, Indianapolis, IN, USA), and the resulting hybridomas were selected in RPMI 1640 medium containing hypoxanthine, aminopterin, and thymidine (Thermo Fisher Scientific, Inc.). Culture supernatants were screened by flow cytometry. The isotype of mAbs were determined using isotype-specific secondary antibodies (SouthernBiothech, Birmingham, AL).

### 2.4 Flow cytometry

Cells were collected following a brief exposure to 0.25% trypsin and 1 mM ethylenediaminetetraacetic acid (Nacalai Tesque, Inc.). The cells were then washed with 0.1% bovine serum albumin (BSA) in phosphate-buffered saline (PBS) and treated with primary mAbs or hybridoma culture supernatants for 30 min at 4 °C. After incubation, cells were treated with Alexa Fluor 488-conjugated anti-mouse IgG (1:2000; Cell Signaling Technology, Inc., Danvers, MA, USA). Fluorescence data were collected using SA3800 Cell Analyzer (Sony Corp.) and analyzed using FlowJo (BD Biosciences, Franklin Lakes, NJ, USA).

### 2.5 Western blot analysis

Cell lysates (10 μg) were boiled in sodium dodecyl sulfate (SDS) sample buffer (Nacalai Tesque, Inc.). Proteins were separated on 5%–20% polyacrylamide gels (FUJIFILM Wako Pure Chemical Corporation) and transferred onto polyvinylidene difluoride (PVDF) membranes (Merck KGaA, Darmstadt, Germany). After blocking with 4% skim milk (Nacalai Tesque, Inc.) in PBS with 0.05% Tween 20, membranes were incubated with 5 μg/mL TrMab-29, 1 μg/mL NZ-1 (an anti-PA tag mAb; rat IgG_2a_, lambda) [27,30], or 1 μg/mL anti-β-actin (clone AC-15; Sigma-Aldrich Corp., St. Louis, MO). Membranes were then incubated with either peroxidase-conjugated anti-mouse immunoglobulins (diluted 1:1000; Agilent Technologies, Inc., Santa Clara, CA, USA) to detect TrMab-29 and anti-β-actin, or with an anti-rat IgG (diluted 1:10,000; Sigma-Aldrich Corp.) to detect NZ-1. Finally, protein bands were detected with a chemiluminescence reagent, ImmunoStar LD (FUJIFILM Wako Pure Chemical Corporation) using a Sayaca-Imager (DRC Co. Ltd., Tokyo, Japan).

### 2.6 Immunohistochemical analysis

Paraffin-embedded tissue sections of a breast cancer tissue array (Cat#T8235721-5, Lot#B104066; BioChain, San Francisco, CA, USA) were autoclaved in EnVision FLEX Target Retrieval Solution High pH (Agilent Technologies, Inc.) for 20 min. After blocking with SuperBlock T20 (Thermo Fisher Scientific, Inc.), tissue sections were incubated with TrMab-29 (5 μg/mL) for 1 h at room temperature and then treated with the EnVision + Kit for mouse (Agilent Technologies Inc.) for 30 min. Color was developed using 3,3′-diaminobenzidine tetrahydrochloride (DAB; Agilent Technologies Inc.) for 2 min. Counterstaining was performed with hematoxylin (FUJIFILM Wako Pure Chemical Corporation). Hematoxylin & eosin (HE) staining (FUJIFILM Wako Pure Chemical Corporation) was performed using consecutive tissue sections. Leica DMD108 (Leica Microsystems GmbH, Wetzlar, Germany) was used to examine the sections and obtain images.

## 3 Results

### 3.1 Establishment of anti-TROP2 mAbs

Two mice were immunized with CHO/PA-TROP2-RAP-MAP cells to develop anti-TROP2 mAbs. Hybridomas showing strong signals from CHO/TROP2-PA cells and weak-to-no signals from CHO–K1 cells were selected using flow cytometry. After additional Western blot and immunohistochemical analyses, we established a novel anti-TROP2 mAb, TrMab-29 (mouse IgG_1_ kappa). Hybridoma cells of TrMab-29 could stably grow and secret mAbs using serum-free medium.

### 3.2 Characterization of TrMab-29

**Fig. 1:**
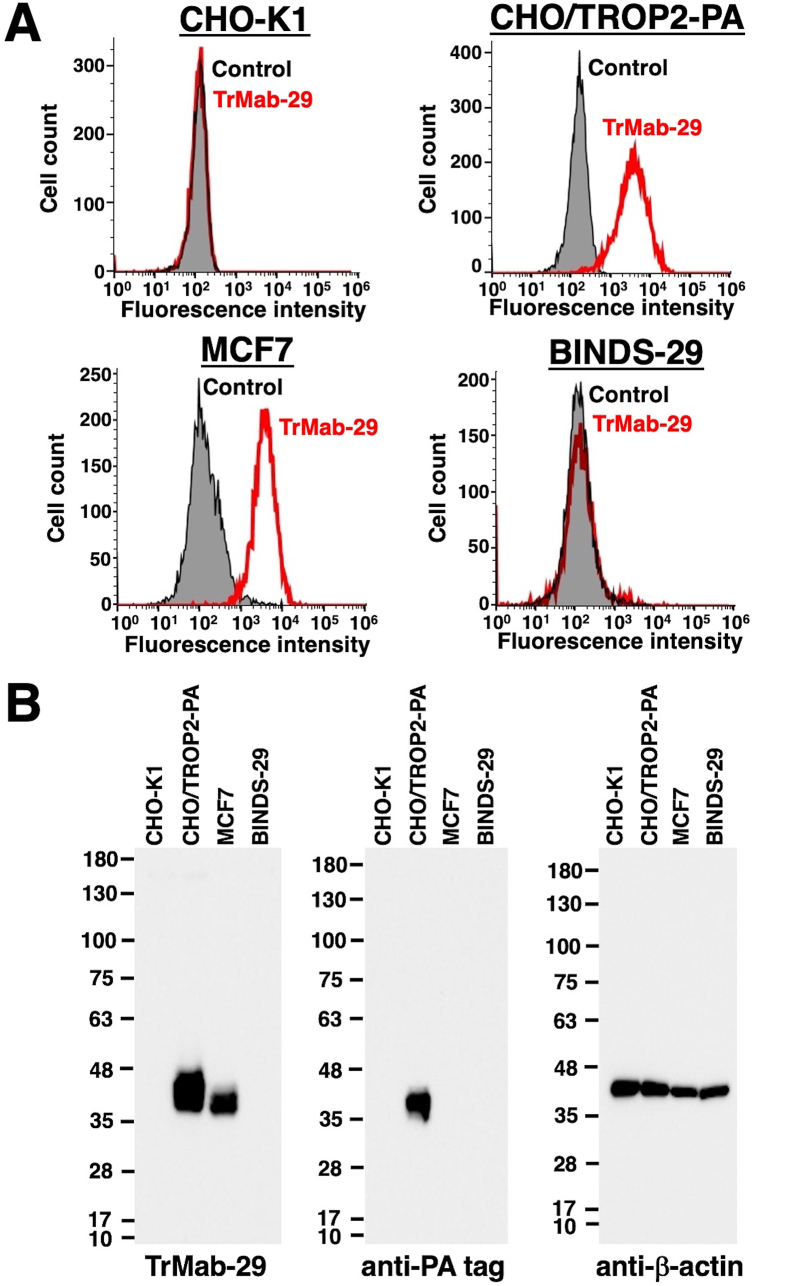
**TROP2 detection by TrMab-29 using flow cytometry and Western blot.** (A) CHO–K1, CHO/TROP2-PA, MCF7, and BINDS-29 (MCF7/TROP2-KO) cells were incubated with TrMab-29 (1 μg/mL; red line) or 0.1% BSA in PBS (gray line) for 30 min, followed by incubation with Alexa Fluor 488-conjugated secondary antibodies. Fluorescence data were collected using a cell analyzer. (B) Detection of TROP2 with TrMab-29 by Western blot analysis. Cell lysates (10 μg) of CHO–K1, CHO/TROP2-PA, MCF7, and BINDS-29 were subjected to SDS-PAGE and transferred to PVDF membranes. Membranes were then incubated with 5 μg/mL of TrMab-29, 1 μg/mL of anti-PA (NZ-1), and 1 μg/mL of anti-β-actin (AC-15), followed by incubation with secondary antibodies and detection.

We first investigated whether TrMab-29 can specifically detect TROP2 by flow cytometry (Fig. 1A). We detected TROP2 in CHO/TROP2-PA cells, but not parental CHO–K1 cells. TrMab-29 also detected endogenous TROP2 on MCF7, a human breast cancer cell line. In contrast, we found that TrMab-29 did not react with TROP2-knockout MCF7 cells (BINDS-29), indicating that TrMab-29 is specific for TROP2.

**Supplementary Fig. 1:**
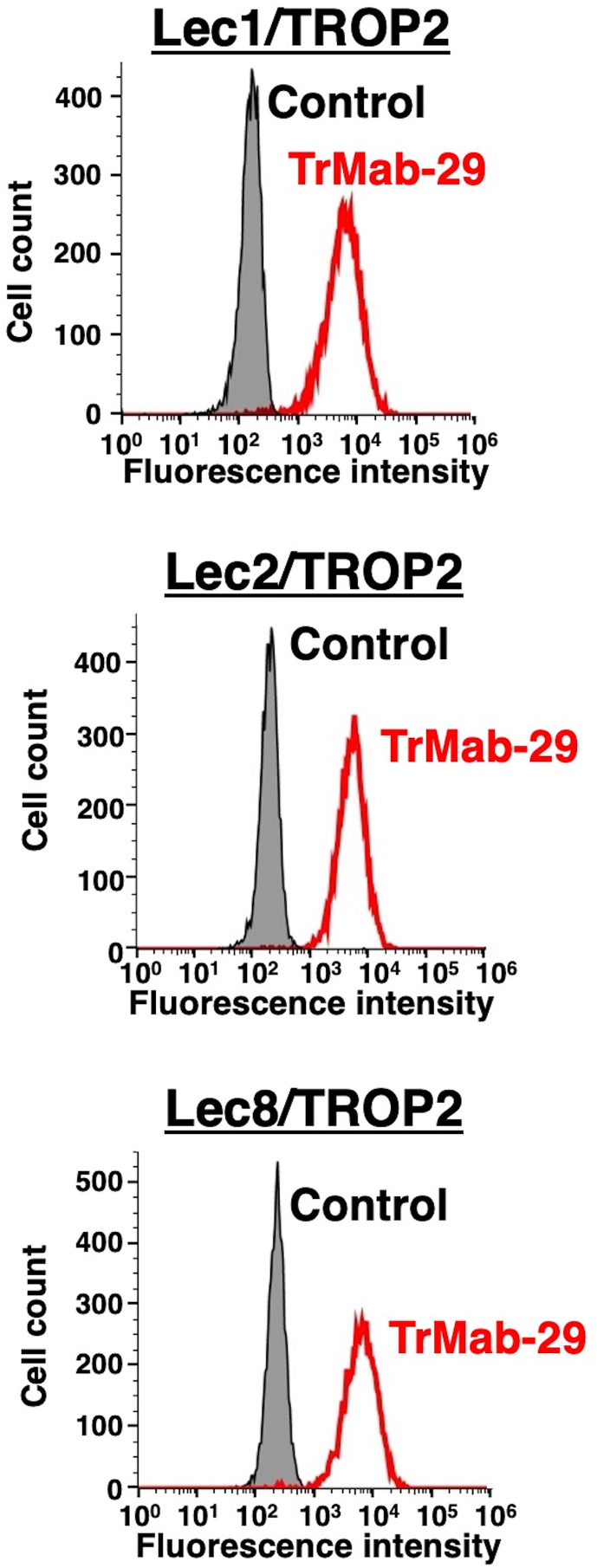
**Characterization of TrMab-29 epitope using glycan-deficient cell lines and flow cytometry.** (A) Lec1/TROP2 (*N*-glycan-deficient), Lec2/TROP2 (sialic acid-deficient), and Lec8/TROP2 (galactose-deficient) cells were incubated with TrMab-29 (1 μg/mL; red line) or 0.1% BSA in PBS (gray line) for 30 min, followed by incubation with Alexa Fluor 488-conjugated secondary antibodies. Fluorescence data were collected using a cell analyzer.

Next, we determined if the TrMab-29 epitope is associated with glycans. We performed flow cytometry using TROP2-transfected glycan-deficient CHO cells, namely *N*-glycan-deficient Lec1, sialic acid-deficient Lec2, and galactose-deficient Lec8 cells. As shown in Supplementary Fig. 1, we found that TrMab-29 reacted with Lec1/TROP2, Lec2/TROP2, and Lec8/TROP2 cells similar to that found with CHO/TROP2-PA cells, indicating that the TrMab-29 epitope is not associated with glycans.

Further, we evaluated the usefulness of TrMab-29 for Western blot analysis. We found a TrMab-29-positive 40 kDa band in lysates from both CHO/TROP2-PA and MCF7 cells (Fig. 1B). In contrast, no band was observed in lysates from CHO–K1 and TROP2-knockout MCF7 (BINDS-29) cells, indicating that TrMab-29 specifically detected both exogenous and endogenous TROP2 in Western blot. An anti-PA tag mAb (NZ-1) also detected a 40-kDa band in the lysates of CHO/TROP2-PA cells.

**Fig. 2:**
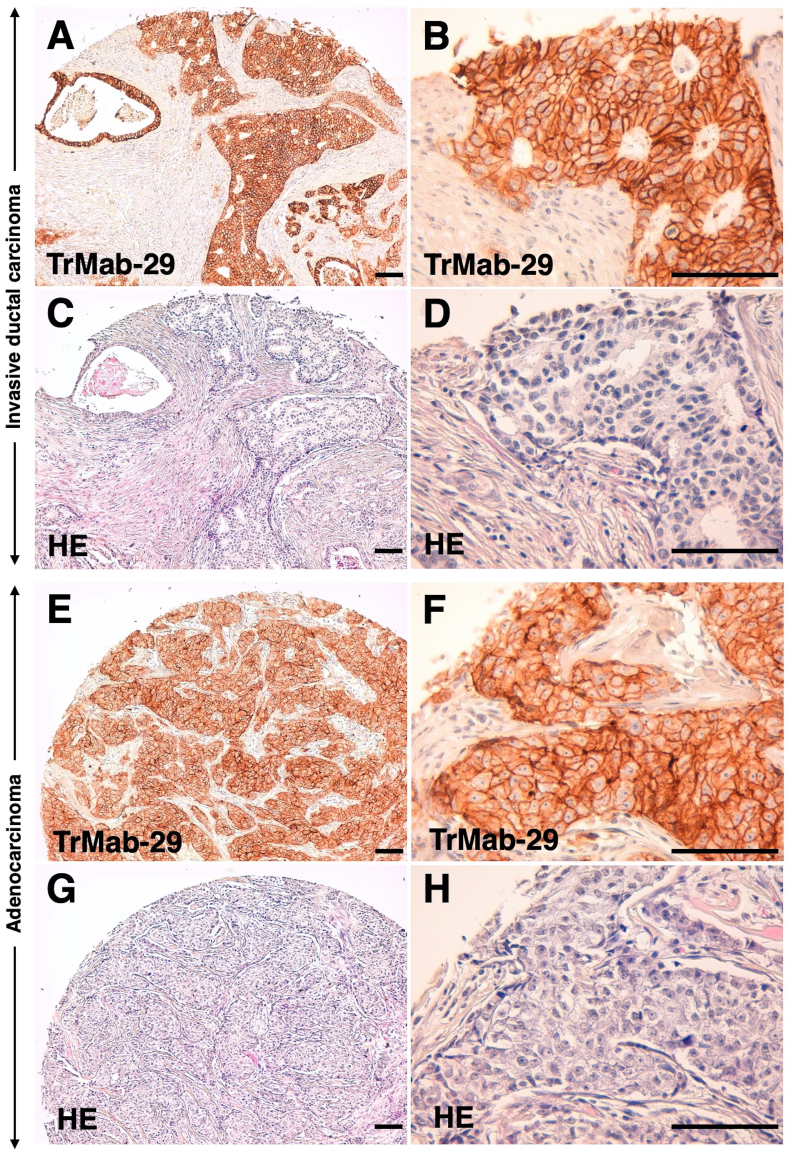
**Detection of TROP2 in breast cancer specimens by immunohistochemical analysis with TrMab-29.** (A, B) Invasive ductal carcinoma tissue sections from patients with breast cancer were incubated with 5 μg/mL TrMab-29 and then treated with the EnVision + Kit. Tissues were counterstained with hematoxylin. (C, D) Hematoxylin and eosin (H&E) staining was performed using consecutive invasive ductal carcinoma tissue sections. (E, F) Adenocarcinoma tissue sections from patients with breast cancer were incubated with 5 μg/mL TrMab-29, followed by treatment with EnVision + Kit. Counterstaining was performed with hematoxylin. (G, H) H&E staining was performed using consecutive adenocarcinoma tissue sections. Scale bar = 100 μm.

Finally, we investigated whether TrMab-29 is suitable for immunohistochemical analysis. As shown in Fig. 2, we found a TrMab-29-positive membrane-staining pattern in invasive ductal carcinoma (Fig. 2A and B) and adenocarcinoma (Fig. 2E and F) tissue sections from patients with breast cancer. Hematoxylin and eosin (H&E) staining of invasive ductal carcinoma sections (Fig. 2C and D) and adenocarcinoma sections (Fig. 2G and H) are also presented for reference. These findings demonstrate that TrMab-29 is well suited for immunohistochemical analysis using paraffin-embedded tissue sections.

## 4 Discussion

Using the CBIS method, in which antigen-expressing cell lines are used for both immunization and screening [15], we have developed many useful mAbs against membrane proteins, including CD19 [16], CD20 [17], CD44 [18], CD133 [15], and PD-L1 [19], for research applications. Of these mAbs, we recently developed clone C_20_Mab-11 to reliably detect CD20 by flow cytometry, Western blot, and immunohistochemical analysis [17]. CD20 has four membrane-spanning domains and only two small extracellular domains that include amino acids 72–80 and 142–182 [31,32]. Although there are several commercially available mAbs that interact with amino acids 142–182 of CD20 and are specifically useful in flow cytometry, there are no available anti-CD20 mAbs that are effective not only in flow cytometry but also in Western blot and immunohistochemical analyses [17], indicating that establishing multipurpose mAbs remains difficult. In this study, we aimed to establish a multipurpose anti-TROP2 mAb using the CBIS method. To that end, we successfully developed a sensitive and specific novel anti-TROP2 mAb (TrMab-29), which can be used in different research applications, such as flow cytometry (Fig. 1A), Western blot (Fig. 1B), and immunohistochemical analyses (Fig. 2).

As a next step, it would be of interest to determine whether TrMab-29 is suitable for use in targeted molecular therapy against TROP2-expressing cancers. Among mouse immunoglobulins, IgG_2a_ and IgG_2b_ can induce antibody-dependent cellular cytotoxicity (ADCC) and complement-dependent cytotoxicity (CDC) [33,34]. As TrMab-29 was determined to belong to the mouse IgG_1_ subclass, which does not exert ADCC/CDC, it should be changed to the IgG_2a_ or IgG_2b_ subclass. Recently, we have focused on various modalities that can be used to promote antibody therapy, including antibody-drug conjugates (ADCs), radioimmunotherapy (RIT), photoimmunotherapy (PIT), and chimeric antigen receptor T-cell (CAR-T) therapy. ADCs include a mAb, a linker, and a “payload,” providing a means in which a cytotoxic drug can be delivered directly to cancer tissues via antibody targeting [35]. RIT is a modality permitting selective internal radiation therapy using radiolabeled mAbs against cancer-associated antigens [36], whereas near-infrared PIT (NIR-PIT) is a novel cancer therapy that combines the specificity of mAbs with directed toxicity induced by a photoabsorber following exposure to NIR light [37]. Alternatively, CAR-T therapy enables T-cells to detect predefined surface antigens on cancer cells independent of major histocompatibility restriction [38]. Following the establishment and characterization of our novel anti-TROP2 mAb described in the current study, we will investigate different treatment modalities, including ADC, RIT, PIT, and CAR-T-cell therapy, to explore TrMab-29-mediated antitumor activities in TROP2-expressing cancers.

## Funding

This research was supported in part by 10.13039/100009619AMED [grant numbers: JP20am0401013, JP20am0101078, JP20ae0101028] (Y·K.).

## Author contributions

Y.S: performed the experiments. M.K.K: designed the experiments. Y.K: designed the experiments. J.T: Formal analysis, analyzed the data. H.H: Formal analysis, analyzed the data. M.S: Formal analysis, analyzed the data. T.A: Formal analysis, analyzed the data. Y.S: Writing – original draft, wrote the manuscript. Y.K: Writing – original draft, wrote the manuscript.

## Declaration of competing interest

The authors declare no conflicts of interest involving this article.
